# Causality between major depressive disorder and functional dyspepsia: a two-sample Mendelian randomization study

**DOI:** 10.3389/fneur.2024.1338153

**Published:** 2024-07-22

**Authors:** Yaming Du, Rui Wang, Xinzi Xu, Junli Wang, Wei Shao, Guohua Chen

**Affiliations:** ^1^Clinical College of Chinese Medicine, Hubei University of Chinese Medicine, Wuhan City, China; ^2^Key Laboratory of Neuropharmacology and Translational Medicine of Zhejiang Province, School of Pharmaceutical Sciences, Zhejiang Chinese Medical University, Hangzhou, China; ^3^Center for General Practice Medicine, Department of Integrated Traditional Chinese and Western Medicine, Zhejiang Provincial People’s Hospital (Affiliated People’s Hospital), Hangzhou Medical College, Hangzhou, Zhejiang, China; ^4^Department of Neurology, Wuhan No. 1 Hospital, Wuhan City, China

**Keywords:** Mendelian randomization, major depressive disorder, functional dyspepsia, causal relationship, disease risk

## Abstract

**Background:**

To investigate the causal relationship between major depression and functional dyspepsia using two-sample Mendelian randomization.

**Methods:**

Data for major depression and functional dyspepsia were obtained from genome-wide association studies. We selected Single Nucleotide Polymorphisms (SNPs) strongly associated with severe depression. Mendelian randomization analysis was conducted using methods such as Inverse-Variance Weighted (IVW), MR-Egger, and Weighted Median Estimator (WME). Sensitivity analysis was performed to assess the robustness of the results.

**Results:**

A total of 31 eligible SNPs were identified as instrumental variables for major depression. IVW analysis indicated a positive causal relationship between the two conditions (*β* = 0.328; SE = 0.137; *p* = 0.017), suggesting that severe depression increases the risk of functional dyspepsia (OR = 1.389; 95% CI: 1.062–1.816). Sensitivity tests showed no evidence of heterogeneity or horizontal pleiotropy (*p* > 0.05).

**Conclusion:**

MR analysis had shown that major depressive disorder is associated with an increased risk of functional dyspepsia.

## 1 Introduction

Functional dyspepsia (FD) is a clinically common gastrointestinal disorder originating from the stomach and duodenum, accounting for approximately 80% of all cases of indigestion. Its primary symptoms include epigastric pain or burning, early satiety during meals, and postprandial fullness. Importantly, these symptoms occur in the absence of any evidence of organic disease that could explain the discomfort (1, 2). FD is a complex, multifactorial disorder associated with gastrointestinal sensory and motor dysfunction, immune dysregulation, and changes in gut microbiota. Studies indicate that 10–30% of the population is affected by FD (3). Risk factors for the onset of FD include being female, smoking, *Helicobacter pylori* infection, acute gastrointestinal inflammation, the use of non-steroidal anti-inflammatory drugs, and mental disorders (4).

Major depressive disorder (MDD) is closely associated with FD (Functional dyspepsia), often co-occurring, but their causal relationship remains unconfirmed. Mendelian randomization (MR) is an epidemiological analysis method that employs genetic variations strongly correlated with exposure or risk factors as instrumental variables (IV) to assess whether there is a causal relationship between exposure or risk factors and clinically relevant outcomes (5). Single nucleotide polymorphisms (SNPs) are the most commonly used genetic variations in MR studies. Because the alleles related to exposure are randomly assigned and are generally unaffected by environmental factors acquired later in life (6), MR analysis not only shares the benefits of randomized allocation in randomized controlled trials (7), but is also less susceptible to biases such as confounding or reverse causality (8). Therefore, the current study aims to investigate the causal relationship between MDD and FD through a two-sample MR analysis.

## 2 Materials and methods

### 2.1 Study design

**Figure 1:**
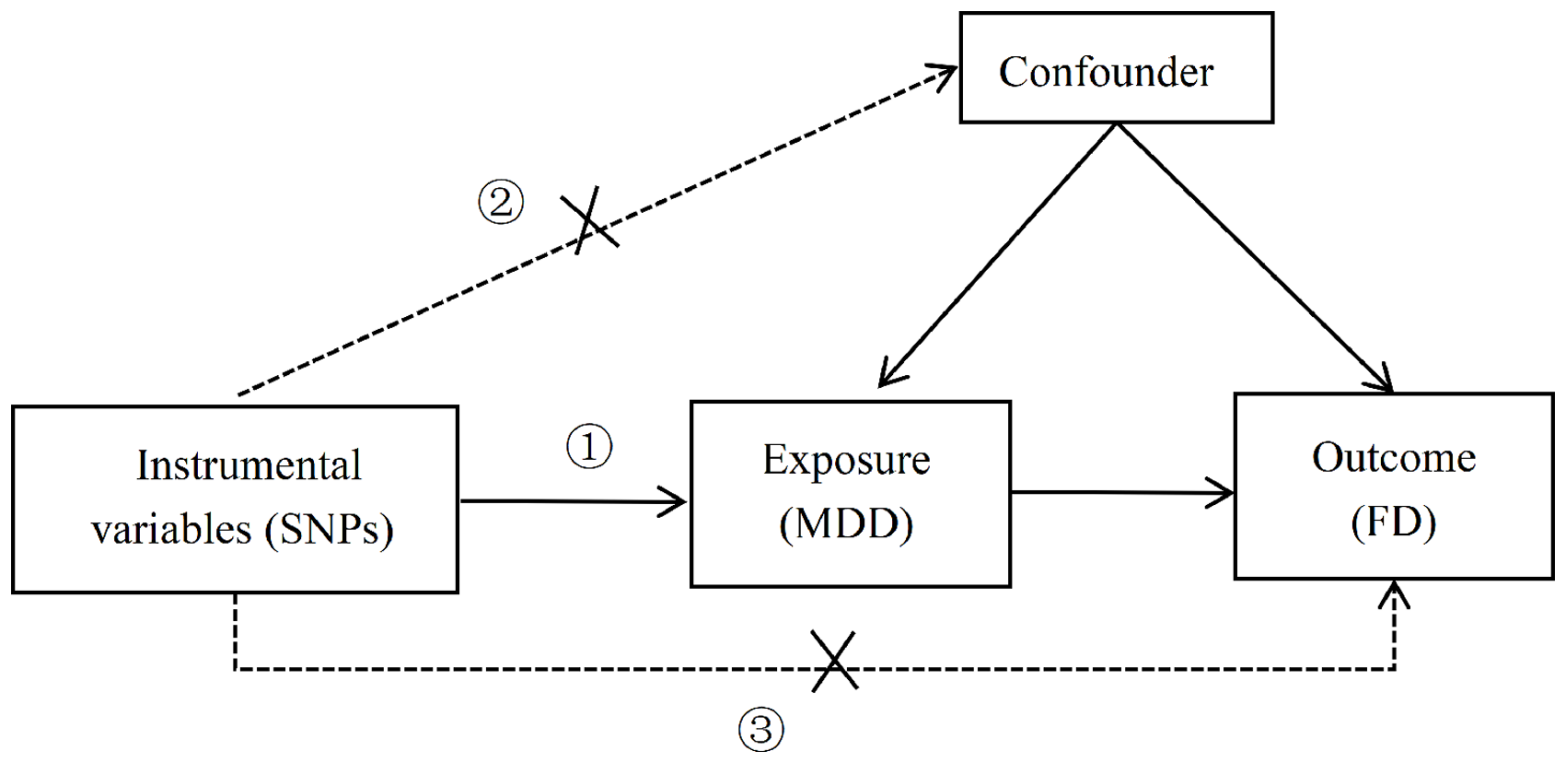
Three core assumptions of MR analysis (①: the correlation hypothesis; ②: the independence hypothesis; ③: the assumption of exclusivity).

Valid instrumental variables (IVs) are crucial for MR (Mendelian Randomization) studies and must adhere to the three core assumptions of MR analysis (6, 8): ① Relevance assumption: the IVs must be strongly correlated with the exposure variable (MDD); ② Independence assumption: the IVs should not be related to any confounders; ③ Exclusion restriction assumption: the IVs must influence the outcome (FD) only through the exposure variable (MDD), and not through any other pathways. See Figure 1.

### 2.2 Data source

The genome-wide association study (GWAS) data for both the exposure (MDD) and the outcome (FD) used in this study were sourced from the website https://gwas.mrcieu.ac.uk/datasets/. The data for MDD was obtained from the Psychiatric Genomics Consortium (PGC) and is based on a 2018 GWAS summary that includes 480,359 participants (135,458 cases and 344,901 controls). The FD data comes from a 2021 GWAS summary and includes a sample size of 194,071, consisting of 4,376 cases and 189,695 controls. Both the MDD and FD samples were of European ancestry. Detailed information can be found in Table 1.

**Table 1:** GWAS data information in the two-sample MR study.

Disease	Sample size	Population	Number of SNPs	Gender	Publication year
MDD	480,359	European	10,000	Mixed Gender	2018
FD	194,071	European	16,380,380	Mixed Gender	2021

### 2.3 Selection of instrumental variables

In this study, suitable SNPs will be selected as instrumental variables (IVs) by adhering to the three core assumptions of MR. First, SNPs with genome-wide significance (*p* < 5 × 10^−8^) will be filtered from the GWAS data for MDD. To control for the impact of linkage disequilibrium (LD), parameters are set at *r*^2^ < 0.001 and a distance of 10,000 kb. PhenoScanner database will be utilized to search the phenotypes of each SNP to rule out the effects of confounding factors (9). To ensure that the selected SNPs are strongly correlated with the exposure, the *F*-statistic will be employed to evaluate the weak instrument effect. An *F*-value greater than 10 will be considered indicative of no weak instrument bias (10).

### 2.4 MR analysis

This study will conduct two-sample MR analysis using the TwoSampleMR package in R version 4.3.1. The primary analytical methods include inverse-variance weighted (IVW), MR-Egger regression, and weighted median estimator (WME) (11). IVW provides accurate causal estimates under the assumption that all variants are valid instrumental variables (12).

### 2.5 Sensitivity analysis

To evaluate the robustness of the conclusions, sensitivity analyses will be performed on the results, which include heterogeneity tests, horizontal pleiotropy tests, and Leave-One-Out analysis. (1) Heterogeneity Tests (13): Cochran’s *Q* test will be used to examine heterogeneity across instrumental variables, to evaluate differences among different SNPs (*p* > 0.05 indicates no heterogeneity). (2) Horizontal Pleiotropy Tests: MR-Egger intercept (13) (when the intercept term in MR-Egger is close to zero, it suggests no horizontal pleiotropy among SNPs; if it diverges significantly from zero, it suggests the presence of horizontal pleiotropy) and MR-PRESSO (14) (Global test *p* > 0.05, indicating no horizontal pleiotropy) will be used to test for horizontal pleiotropy. (3) Leave-One-Out Analysis: each SNP will be sequentially removed to observe whether the results change upon omission. If the removal of SNPs one by one does not significantly impact the results, this suggests the analysis is robust. All statistical tests with *p* < 0.05 are considered statistically significant.

## 3 Results

### 3.1 Instrumental variables associated with MDD

After screening with the criteria of *p* < 5 × 10^−8^ and removing linkage disequilibrium (LD), an initial set of 36 SNPs was obtained. Confounders were eliminated through the PhenoScanner database, and the remaining SNPs were matched with the outcome dataset and palindromic sequences were removed, resulting in 31 SNPs being selected as instrumental variables for MDD. Calculation shows that the *F*-values of all included SNPs are consistently greater than 10, suggesting that the influence of weak instrumental variables is unlikely.

### 3.2 MR analysis results

**Figure 2:**
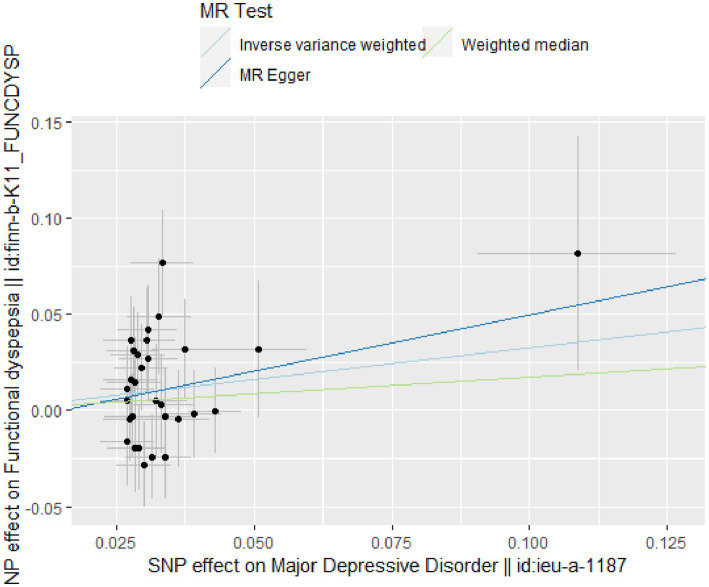
Scatter plot of two-sample MR analysis.

**Figure 3:**
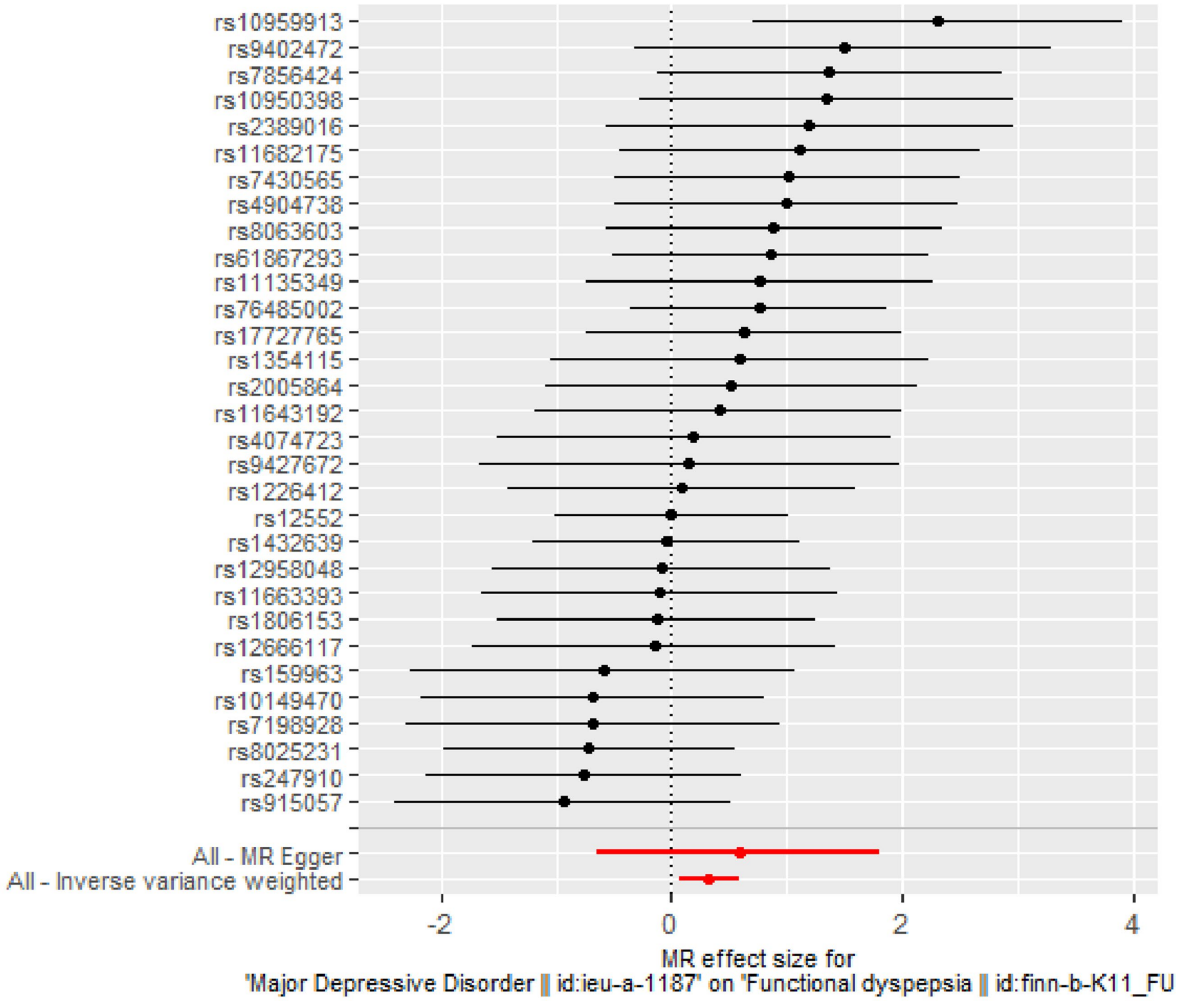
Forest plot of two-sample MR analysis.

The IVW analysis indicates a positive causal relationship between MDD and FD (*β* = 0.328; SE = 0.137; *p* = 0.017). MDD is associated with an increased risk of FD (OR = 1.389; 95% CI: 1.062–1.816), as shown in Figures 2, 3.

### 3.3 Sensitivity analysis

**Figure 4:**
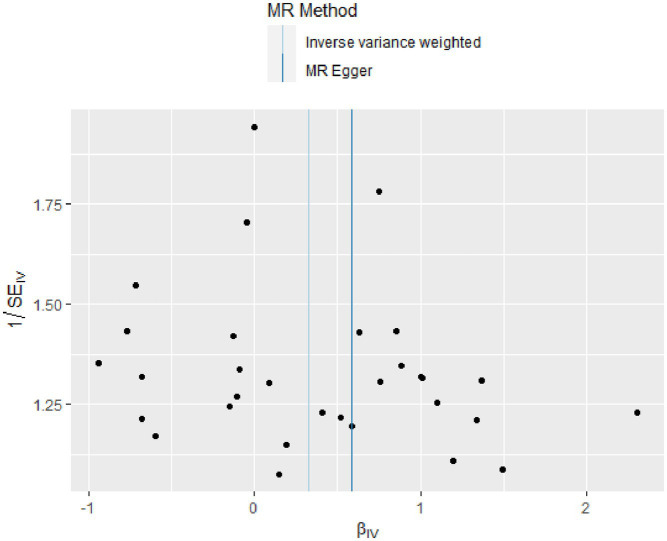
Funnel plot of heterogeneity test results in two-sample MR analysis.

**Figure 5:**
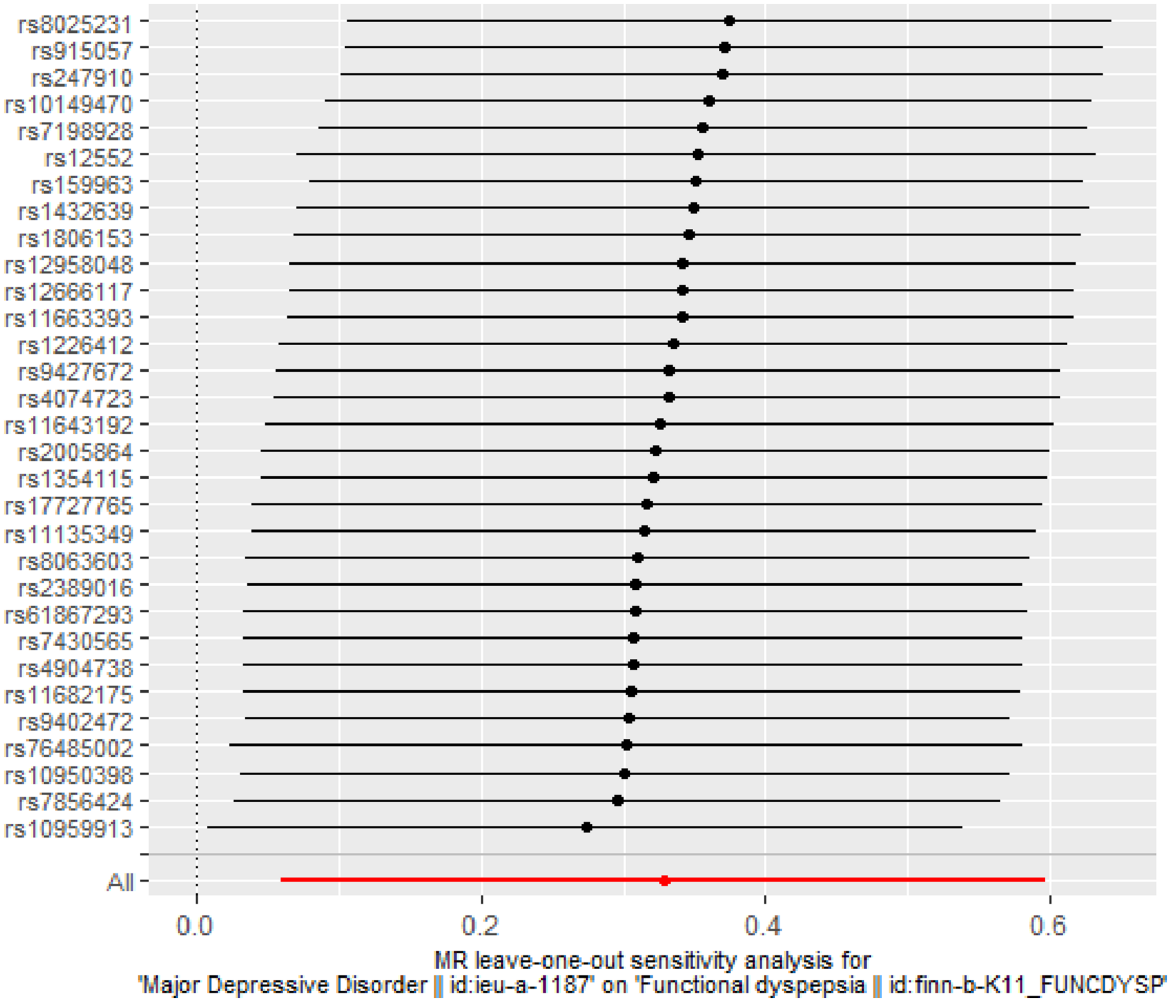
Leave-One-Out sensitivity analysis results of two-sample MR analysis.

The *Q*-tests for both IVW (*p* = 0.391) and MR-Egger regression (*p* = 0.351) show no heterogeneity among the included SNPs, as depicted in Figure 4. The MR-Egger intercept is close to zero (MR-Egger intercept = −0.008, *p* = 0.679), and the MR-PRESSO Global Test *p* = 0.484 > 0.05, indicating the absence of horizontal pleiotropy. The Leave-one-out method did not identify any SNPs that significantly influenced the estimates, suggesting that the analysis results are robust, as depicted in Figure 5.

## 4 Discussion

Major Depressive Disorder (MDD) is a prevalent and severe mental illness that increases the risk of various somatic diseases. Past cohort studies and meta-analyses have indicated a correlation between depressive symptoms and Functional Dyspepsia (FD). A matched cohort study based on 40,394 participants showed that depression is associated with an increased risk of developing FD, with an adjusted HR of 2.16 and a 95% CI of 1.93 to 2.41 (15). Another meta-analysis that included 23 studies also demonstrated a significant association between FD and depression (16). However, the evidence for this relationship remains inconclusive due to confounding factors and reverse causality often found in observational studies. This study identifies a link between MDD and FD using two-sample Mendelian Randomization (MR) methods, establishing MDD as a risk factor for FD.

The relationship between Major Depressive Disorder (MDD) and gastrointestinal diseases is distinct from other chronic conditions, due to the unique interaction between the central nervous system and the gastrointestinal tract, also known as the brain-gut axis. The interplay between MDD and Functional Dyspepsia (FD) is complex, involving physiological, neuroendocrine, immunological, and gut microbiota factors. Key aspects include: ① Dysregulation of the Hypothalamic-Pituitary-Adrenal (HPA) Axis: research indicates that HPA axis dysfunction is observed in patients with psychiatric disorders and mood disorders. In patients with MDD, abnormalities in the HPA axis manifest as increased secretion and reactivity of cortisol, along with elevated levels of corticotropin-releasing hormone (CRH) in cerebrospinal fluid (17). Activation of CRH receptors can interact with various CRF receptor subtypes, leading to inhibited gastric emptying and altered colonic motility (18). Furthermore, cortisol receptors are expressed in various intestinal cells, indicating that cortisol has a direct impact on the gastrointestinal tract (19). ② Alterations in Gut Microbiota: studies have shown a link between MDD and changes in gut microbial abundance (20). Research by Jiang et al. found a reduction in Faecalibacterium in MDD patients, which negatively correlated with the severity of depressive symptoms (21). ③ Inflammatory Response: research has indicated that MDD is associated with systemic immune activation, leading to increased levels of pro-inflammatory cytokines (22). These elevated cytokines may compromise the intestinal mucosal barrier, contributing to the pathogenesis of FD. In addition, MDD can also cause gastrointestinal symptoms by affecting substances like brain-gut peptides and serotonin.

This study is the first to definitively establish the relationship between Major Depressive Disorder (MDD) and Functional Dyspepsia (FD) using Mendelian Randomization (MR) methods. The SNPs used in the study showed strong correlation with MDD (*p* < 5 × 10^−8^, *F* > 10) and were pruned for linkage disequilibrium with the condition *r*^2^ < 0.001, kb = 10,000 to ensure their independence. In terms of results, the Inverse Variance Weighted (IVW) method showed significant results, while MR-Egger and the Weighted Median Estimator (WME) methods did not. However, no pleiotropy or heterogeneity was observed, and the beta values obtained from other methods were consistent in direction with the IVW method. Thus, we can infer a positive causal relationship between MDD and FD.

Nevertheless, this study has some limitations. The population included was of European descent, so the conclusions may not be generalizable to other ethnic groups and further studies are required to explore this. Moreover, since the samples in this study came from the public database, exposure variables could not be stratified according to gender or age, so the effect of age or sex on this causal relationship cannot be determined.

In summary, this study used a two-sample MR analysis method to assess whether MDD has an impact on the onset of FD. Genetic evidence suggests that individuals with MDD are at an increased risk of developing FD, identifying it as a genetic risk factor for the onset of FD. This offers clinical insights, as many depressive patients present with gastrointestinal discomfort as their primary symptom upon consultation, often masking the underlying etiology and leading to misdiagnoses, resulting in suboptimal treatment outcomes. Concurrently, when addressing the pathogenesis of FD, one should fully consider the neuropsychological status of the patient.

## Data availability statement

Publicly available datasets were analyzed in this study. This data can be found at: Medical Research Council (MRC) Integrative Epidemiology Unit (IEU) OpenGWAS project, https://gwas.mrcieu.ac.uk/datasets/.

## Ethics statement

Ethical review and approval was not required for the study on human participants in accordance with the local legislation and institutional requirements. Written informed consent from the patients/participants or patients/participants' legal guardian/next of kin was not required to participate in this study in accordance with the national legislation and the institutional requirements.

## Author contributions

YD: Writing – original draft, Writing – review & editing. RW: Data curation, Writing – original draft, Writing – review & editing. XX: Data curation, Methodology, Writing – review & editing. JW: Writing – review & editing. WS: Supervision, Writing – review & editing. GC: Supervision, Writing – review & editing.
