# Supplementary material for: Causality between major depressive disorder and functional dyspepsia: a two-sample Mendelian randomization study
Source: Front Neurol. 2024 Jul 22;15:1338153. doi: 10.3389/fneur.2024.1338153 (PMC11298389; doi:10.3389/fneur.2024.1338153)
Supplement: Supplementary file 1 [file Data_Sheet_1.docx]

Supplementary Material

Causality between major depressive disorder and functional dyspepsia: a two-sample Mendelian randomization study

**Supplementary tables:**

Supplementary table1: SNPs (n=36) of MDD.

| SNP | effect_allele | other_allele | beta | se | pval |
| --- | --- | --- | --- | --- | --- |
| rs159963 | A | C | -0.0270013 | 0.0049 | 3.19E-08 |
| rs1432639 | A | C | 0.0389995 | 0.005 | 4.55E-15 |
| rs2389016 | T | C | 0.0305001 | 0.0053 | 1.02E-08 |
| rs9427672 | G | A | 0.0320997 | 0.0058 | 3.12E-08 |
| rs1226412 | T | C | 0.0332026 | 0.0059 | 2.38E-08 |
| rs76485002 | G | A | -0.108702 | 0.018 | 1.60E-09 |
| rs11682175 | C | T | 0.0281012 | 0.0048 | 4.68E-09 |
| rs7430565 | A | G | -0.0288008 | 0.0048 | 2.87E-09 |
| rs34215985 | G | C | 0.037297 | 0.0063 | 3.13E-09 |
| rs11135349 | C | A | 0.0293979 | 0.0048 | 1.09E-09 |
| rs247910 | G | A | 0.031501 | 0.0049 | 1.07E-10 |
| rs1363104 | G | C | -0.0314018 | 0.0048 | 7.38E-11 |
| rs9402472 | A | G | 0.0326995 | 0.0059 | 2.78E-08 |
| rs10950398 | A | G | 0.0274984 | 0.0049 | 2.55E-08 |
| rs12666117 | A | G | 0.0274011 | 0.0048 | 1.35E-08 |
| rs1354115 | A | C | 0.0275957 | 0.0049 | 2.37E-08 |
| rs10959913 | G | T | -0.0333961 | 0.0057 | 5.06E-09 |
| rs7856424 | T | C | -0.0306035 | 0.0053 | 8.48E-09 |
| rs61867293 | T | C | -0.0374008 | 0.0061 | 6.97E-10 |
| rs1806153 | T | G | 0.0361005 | 0.0059 | 1.18E-09 |
| rs4074723 | C | A | 0.0270013 | 0.0049 | 3.12E-08 |
| rs12552 | G | A | -0.0428966 | 0.0048 | 6.07E-19 |
| rs10149470 | G | A | 0.0289964 | 0.0049 | 3.05E-09 |
| rs4904738 | C | T | 0.0289037 | 0.0049 | 2.57E-09 |
| rs915057 | G | A | 0.0299954 | 0.0049 | 7.61E-10 |
| rs2005864 | T | C | 0.0281987 | 0.0049 | 6.73E-09 |
| rs8025231 | C | A | 0.0338981 | 0.0048 | 2.36E-12 |
| rs7198928 | C | T | -0.0284028 | 0.005 | 1.00E-08 |
| rs11643192 | A | C | 0.0270021 | 0.0049 | 3.36E-08 |
| rs8063603 | A | G | -0.0307995 | 0.0053 | 6.86E-09 |
| rs17727765 | C | T | 0.0507987 | 0.0088 | 8.51E-09 |
| rs12958048 | G | A | -0.0338022 | 0.0051 | 3.61E-11 |
| rs11663393 | A | G | 0.0278 | 0.0049 | 1.65E-08 |
| rs62099069 | T | A | 0.0278955 | 0.0049 | 1.31E-08 |
| rs5758265 | A | G | 0.0310044 | 0.0054 | 7.55E-09 |
| rs6905391 | A | G | -0.0442968 | 0.0069 | 1.35E-10 |

Supplementary table 2: The results of MR ananlysis.

| exposure | outcome | method | nsnp | b | se | pval |
| --- | --- | --- | --- | --- | --- | --- |
| Major Depressive Disorder | Functional dyspepsia | MR Egger | 31 | 0.584199389 | 0.627060593 | 0.359210523 |
| Major Depressive Disorder | Functional dyspepsia | Weighted median | 31 | 0.173518287 | 0.190470154 | 0.362295437 |
| Major Depressive Disorder | Functional dyspepsia | Inverse variance weighted | 31 | 0.328262889 | 0.136918615 | 0.016507213 |
| Major Depressive Disorder | Functional dyspepsia | Simple mode | 31 | 0.751220246 | 0.413712197 | 0.079407626 |
| Major Depressive Disorder | Functional dyspepsia | Weighted mode | 31 | 0.054332921 | 0.361530478 | 0.881544989 |
